# Study on the Shear Mechanical Behaviour of Asphalt Aggregate Interface Under the Combined Effect of Temperature and Asphalt Film Thickness

**DOI:** 10.3390/ma19204400

**Published:** 2026-10-19

**Authors:** Lina Wang, Chengchao Guo, Mansheng Dong, Bo Wang, Yuanchao Dong, Chao Zhang, Yangming Gao, Xiaodong Yang

**Affiliations:** 1 School of Water Conservancy and Transportation, Zhengzhou University, Zhengzhou 450001, China; 2 School of Civil Engineering, Sun Yat-sen University and Southern Marine Science and Engineering Guangdong Laboratory (Zhuhai), Zhuhai 519082, China; 3 School of Automotive and Transportation Engineering, Hefei University of Technology, Hefei 230002, China; 4 Built Environment and Sustainable Technologies (BEST) Research Institute, Liverpool John Moores University, Liverpool L3 3AF, UK; 5 School of Transportation Science and Engineering, Harbin Institute of Technology, Harbin 150096, China

**Keywords:** SBS modified asphalt, asphalt aggregate interface, shear test, cohesion failure, adhesion failure

## Abstract

The interface between asphalt and aggregate acts as a key link in load transfer and damage development; its shear mechanical behaviour has a decisive influence on the fatigue cracking and durability of bituminous pavements. In this study, SBS-modified asphalt and limestone aggregate were selected. By precisely controlling the moulding load, asphalt film thicknesses ranging from 23 μm to 219 μm were achieved. Systematic shear tests were conducted within a temperature range of 10 °C to 40 °C to systematically investigate the evolution of interfacial shear strength, stress–displacement curve characteristics, shear fracture energy and failure modes. Two primary failure modes were identified: adhesive failure, defined as debonding at the asphalt–aggregate interface with minimal residual asphalt on the aggregate surface; and cohesive failure, defined as internal rupture within the asphalt film with asphalt residue covering both fracture surfaces. The results indicate that temperature has a significant deteriorating effect on interfacial shear strength; as temperature rises, shear strength decays rapidly at first and then more slowly before gradually approaching zero, whilst shear fracture energy decreases monotonically, with the rate of decay being more pronounced at lower temperatures; the thickness of the bituminous film exerts a monotonically decreasing influence on interfacial shear strength, whilst shear fracture energy remains at a relatively high level within the intermediate film thickness range; bituminous films that are either too thin or too thick are detrimental to the effective dissipation of interfacial energy. An analysis of interfacial failure modes reveals that, under low-temperature conditions, failure occurs primarily at the interface between the asphalt and the aggregate, manifesting as adhesion failure; under high-temperature conditions, the failure path shifts to within the asphalt film, manifesting as cohesion failure. An increase in asphalt film thickness promotes a shift towards cohesion failure, whilst a decrease in film thickness tends to favour adhesion failure; between these two extremes, a coupled and competitive relationship is observed. Based on interfacial thermodynamics and phase transition theory, this study provides an interpretation of how temperature governs the migration of failure modes through alterations in asphalt cohesion, intermolecular forces and interfacial surface tension, and reveals the pivotal role of film thickness in restructuring the distribution of interfacial weak points by regulating the ratio of structural asphalt to free asphalt.

## 1. Introduction

Asphalt mixtures are multi-phase, heterogeneous composite materials comprising a mineral aggregate matrix, an asphalt binder, and the interfacial transition zone between the two [1,2,3]. Their macroscopic mechanical behaviour, particularly their resistance to fatigue cracking and permanent deformation, is not a simple weighted average of the properties of their individual components, but is largely governed by the load transfer efficiency and energy dissipation mechanisms at the interface between the aggregate and the bituminous binder [4,5]. As the essential bridge through which stress is transmitted from the bituminous matrix to the rigid aggregate skeleton, the mechanical integrity of the interfacial layer directly determines the pavement structure’s ability to withstand the coupled effects of heavy traffic loads and multiple environmental factors throughout its service life [6,7,8,9]. Retrospective analyses of numerous cases of early-stage pavement damage indicate that cracks typically originate in the weak interface zone at the edges of aggregates; they subsequently propagate and penetrate the pavement under repeated traffic loading, ultimately leading to functional and structural failure of the pavement structure [10,11,12,13]. Consequently, gaining an in-depth understanding of the shear mechanical behaviour of the asphalt-aggregate interface and the physical nature of its failure is one of the key issues in advancing the theoretical framework for the design of durable asphalt pavements.

With regard to the adhesion properties between aggregates and asphalt, the academic community has established a multi-dimensional, multi-scale theoretical framework [14,15,16]. Adhesion theories based on solid mechanics and surface chemistry primarily encompass physical adsorption mechanisms as well as chemical adsorption mechanisms based on the oriented adsorption of polar molecules and the electrostatic effects of the double layer. Surface free energy theory quantifies the wetting and adhesion work of asphalt on aggregate surfaces from a thermodynamic perspective. At the level of characterisation techniques, methods ranging from traditional water boiling and photoelectric colorimetry to more sophisticated pull-out tests, dynamic shear rheological tests and scanning electron microscopy (SEM) observations of microstructures have all revealed, from different perspectives, the energy thresholds and morphological characteristics associated with interface failure [17,18,19]. However, it must be noted that the core focus of existing research paradigms has long centred on the static characterisation of adhesion properties and the analysis of single-variable effects, whilst lacking in-depth theoretical consideration of the dynamic failure processes of interfacial phases under external forces and the competitive mechanisms of failure modes.

As a typical thermoplastic polymeric material, asphalt exhibits a high degree of sensitivity to temperature changes in its mechanical behaviour; this temperature-sensitive characteristic profoundly influences the stress response of the interfacial phase [20,21]. When the temperature decreases, the cohesive strength of asphalt increases, causing the material to exhibit brittle behaviour; when the temperature rises, the viscosity of asphalt drops sharply, resulting in increased fluidity. However, the effect of temperature on the interfacial phase exhibits a marked duality: on the one hand, temperature changes directly alter the viscoelastic modulus and cohesive strength of the asphalt itself; on the other hand, by influencing molecular thermal motion, temperature weakens or strengthens the physicochemical bonding forces between the asphalt and the aggregate surface, thereby altering the interfacial adhesion strength. Existing studies often examine the degradation patterns of the bituminous binder’s own properties in isolation and extrapolate these directly to interfacial behaviour. Within a range of tens of micrometres close to the aggregate surface, the asphalt is constrained by surface force fields to form a high-density, high-cohesive structured asphalt, whilst the region distant from the interface consists of ‘free asphalt’ with relatively weaker mechanical properties. The vast disparity in mechanical response between these two states means that the overall failure behaviour of the interfacial phase cannot be described by a single homogenisation parameter.

Against this backdrop, the mechanical significance of the asphalt film thickness has long been under-recognised [22,23]. In traditional bituminous mixture design, the asphalt film thickness is typically regarded merely as an average volumetric indicator used to estimate the specific surface area of aggregates and the optimum asphalt content. However, from the perspective of interfacial mechanics, changes in film thickness essentially reconfigure the distribution field of cohesive forces within the interfacial phase: when the film is thin, bound asphalt dominates, and the weak points at the interface are concentrated in the physicochemical adsorption layer on the aggregate surface, with failure tending to occur along the interface; when the film thickness increases, the proportion of free asphalt rises significantly, and defects and stress concentrations within the asphalt film become the controlling factors, with failure paths more likely to shift to the interior of the asphalt. More notably, temperature and film thickness do not act independently but exhibit a coupled interaction; under high-temperature conditions, the severe softening of free asphalt drastically amplifies the film thickness effect, causing a reversal in the failure mode [24]. However, this coupled mechanism has been virtually overlooked in the existing literature, with a lack of systematic experimental data to support it and theoretical models to explain it.

In view of this, this paper systematically investigates the shear mechanical behaviour at the asphalt-aggregate interface. The study utilised SBS-modified asphalt and limestone aggregate to design and prepare aggregate-asphalt–aggregate shear specimens. By precisely controlling the moulding load, asphalt film thicknesses ranging from 23 μm to 219 μm were achieved, and shear tests were conducted across the full temperature range from 10 °C to 40 °C. The study not only focuses on the evolution characteristics of peak shear strength and stress–displacement curves, but also introduces shear fracture energy as an energy criterion for interfacial failure. The aim is to reveal the migration patterns of interface weak points and the critical conditions for the competitive transition between failure modes under the combined influence of temperature and film thickness, and to elucidate the mechanisms governing the evolution of the mechanical equilibrium state of the structured asphalt–free asphalt system with respect to temperature and film thickness.

## 2. Materials and Methods

### 2.1. Test Materials

The test asphalt used was SBS-modified asphalt. SBS is a styrene-based thermoplastic elastomer, specifically a styrene–butadiene–styrene triblock copolymer, which exhibits excellent performance at both high and low temperatures and temperature-sensitive properties, and is widely used in pavement structural layers [25,26]. Testing was carried out on the three key performance indicators of the SBS-modified asphalt, and the results are shown in Table 1.

Limestone exhibits good adhesion with asphalt, which contributes to the excellent road performance of bituminous mixtures. Limestone, consisting primarily of calcium carbonate and exhibiting alkaline properties, was selected as the test aggregate. Tests were conducted on the Los Angeles abrasion value, crushing value, water absorption and apparent relative density of the limestone, and the results for each parameter are shown in Table 2.

### 2.2. Sample Preparation and Testing

The test specimens consisted of stone-asphalt-stone sandwich specimens. Limestone columns were used as the test aggregate, and the specific preparation process was as follows: limestone slabs 40 mm in height were selected, some of which were cut into 20 mm-high limestone slabs. One side of each limestone slab was uniformly ground using 120-grade SIC abrasive particles to ensure consistent surface roughness; the surface roughness of the ground slabs was 37.8 μm. A core drill was then used to extract the treated slabs, yielding limestone columns of two sizes: 19 mm in diameter and heights of 40 mm and 20 mm, respectively. The extracted columns were boiled in water for 15 min to remove surface dust, then placed in an oven at 80 °C for 5 h before being removed and cooled to room temperature, resulting in properly treated limestone columns.

The specimen preparation procedure was conducted as follows. First, the SBS-modified asphalt was placed in an oven for 60 min to achieve a fully fluid state. One flat end of a prepared 40 mm-high stone column was dipped into the molten asphalt to a controlled depth of approximately 2 mm, ensuring uniform wetting of the contact surface. The asphalt-coated end was then gently pressed against the flat end of a prepared 20 mm-high stone column to form a sandwich assembly. The assembly was immediately transferred to a custom-designed moulding fixture, which consists of a loading bracket, a specimen clamping device, and a weight platform. The fixture ensures that the applied load is transmitted axially through the specimen, maintaining alignment between the two stone columns.

The loaded fixture was placed in a temperature-controlled oven and heated at a controlled rate of 1.5 °C/min. Upon reaching 80 °C, the temperature was maintained for 3 min to allow the asphalt to flow and redistribute under the applied load, reaching equilibrium thickness. The fixture was then removed from the oven and allowed to cool to room temperature under the sustained load for 2 h to prevent spring-back and ensure dimensional stability. After cooling, the specimens were carefully removed from the fixture, and excess asphalt extruded beyond the aggregate contact area was trimmed using a heated blade. The moulded specimens were then cured for 24 h to allow full relaxation of residual stresses. Aggregate–asphalt–aggregate sandwich specimens with varying asphalt film thicknesses were obtained by adjusting the load weight.

**Figure 1:**
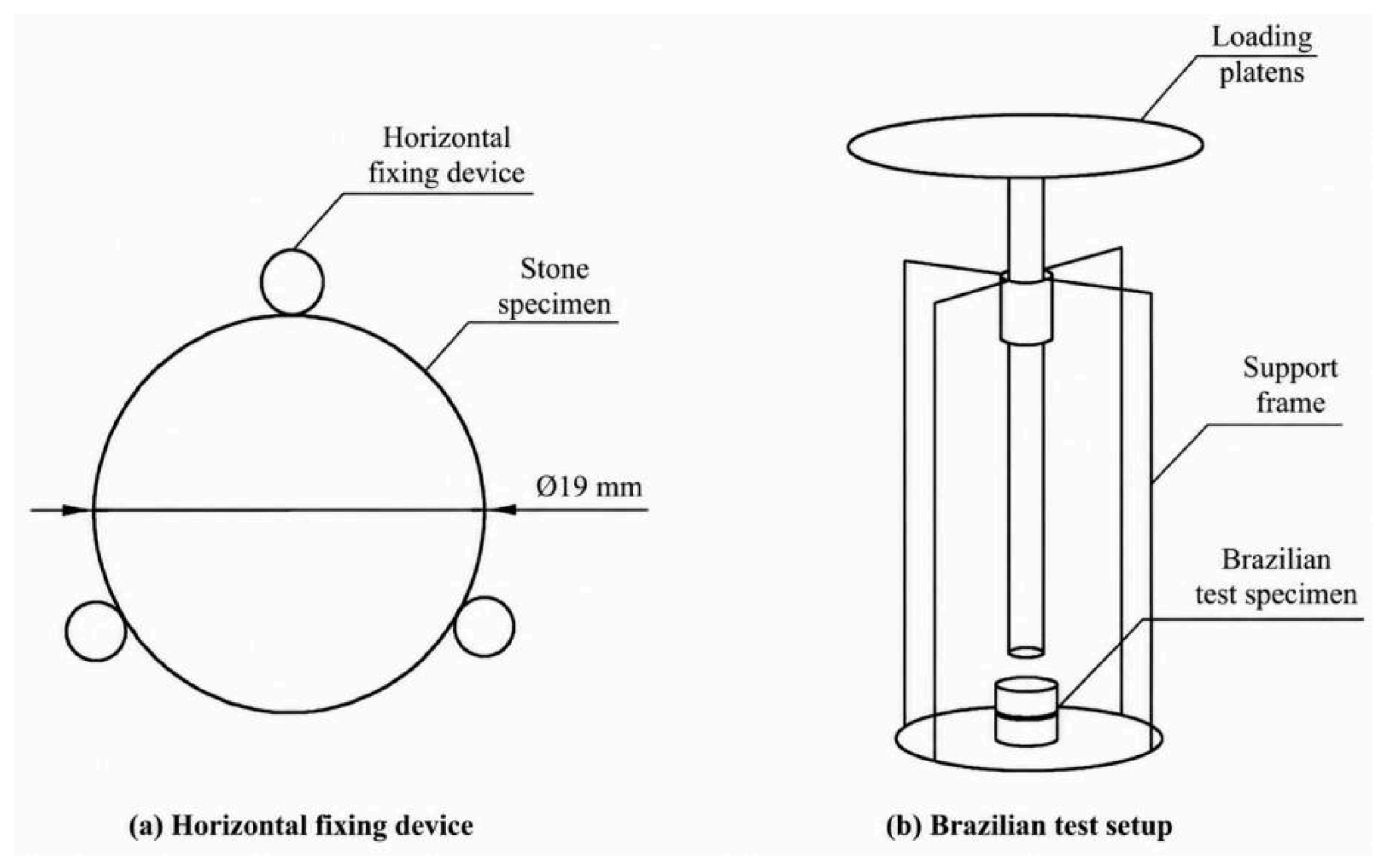
Diagram of fixture design.

This experimental study investigates the relationship between the thickness of the bituminous film and the shear behaviour of bituminous-aggregate mixtures. When preparing sandwich specimens, controlling the thickness of the bituminous film is a key factor. To obtain bituminous-aggregate specimens with different bituminous film thicknesses, a moulding jig for these specimens was designed. The apparatus comprises a loading bracket, a specimen clamping device and weights, amongst other components; the jig design is shown in Figure 1.

Place the asphalt in an oven at 90 °C to soften it. Dip one side of a prepared 40 mm high stone column into the softened asphalt, then gently press it against one side of a prepared 20 mm high stone column to form a sandwich specimen. After cooling to room temperature, remove any excess asphalt from the surface and secure the sandwich specimen in a fixture. Place them in the oven, apply weights, and control the oven temperature. Begin heating from 30 °C until the temperature reaches 80 °C, at which point the timer is started. After 3 min, remove the clamps and specimens, allow them to cool to room temperature, and remove any excess asphalt from the surface to complete the specimen formation. The moulded specimens were cured for 24 h at a room temperature of 25 °C. Aggregate–asphalt–aggregate sandwich specimens with varying asphalt film thicknesses were obtained by adjusting the load weight.

After the specimens had been moulded, the cross-sections of the sandwich specimens were examined under a scanning electron microscope at different moulding loads, and the thickness of the bituminous film was measured. The moulding loads and bituminous film thicknesses are shown in Table 3.

**Figure 2:**
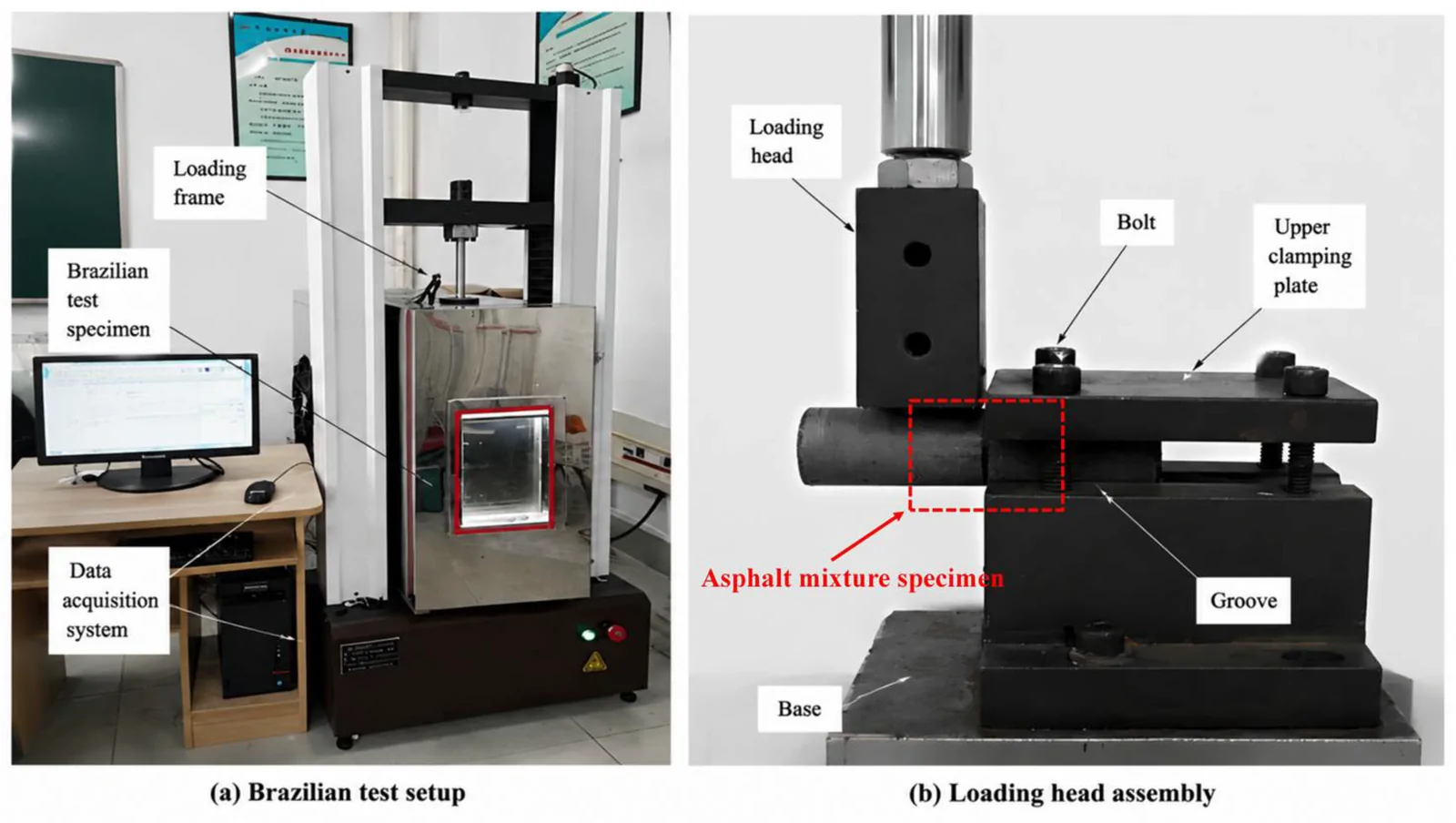
Microcomputer-controlled universal electronic testing machine.

The shear test was carried out on a microprocessor-controlled universal electronic testing machine with temperature control functionality; the instrument is shown in Figure 2. The test setup primarily comprises the universal testing machine, a computer and a temperature chamber.

Shear tests were conducted at a shear rate of 5 mm/min on asphalt films with thicknesses of 219 μm, 129 μm, 61 μm, 34 μm and 23 μm. In summary, the 219 μm thickness was tested at 10, 15, 20, and 25 °C; the 129 μm thickness was tested at 10, 15, 20, 25, 30, and 35 °C; and the 61 μm, 34 μm, and 23 μm thicknesses were tested at 15, 20, 25, 30, and 35 °C. For each temperature–film thickness combination, five (5) independent replicate specimens were tested (*n* = 5). The tests yielded stress–displacement curves.

The mean stress–displacement curves presented in the figures were obtained as follows. For each test condition, five independent replicate specimens were tested (*n* = 5). The individual stress–displacement curves from each replicate were interpolated using a cubic spline method and resampled at uniform displacement intervals of 0.01 mm. At each displacement increment, the mean shear stress was calculated as the arithmetic average of the five replicate values, and the standard deviation was calculated accordingly. The peak shear strength for each replicate was identified as the maximum stress value, and the corresponding displacement was recorded as the peak displacement. The mean peak shear strength and mean peak displacement were then calculated from the five replicate values, along with their standard deviations and coefficients of variation. The mean curves shown in the figures represent these point-by-point averaged stress–displacement relationships, with error bars indicating ±1 standard deviation.

## 3. Results and Discussion

### 3.1. Effect of Temperature on Shear Mechanical Properties of Asphalt Aggregate Interface Phase

#### 3.1.1. Effect of Temperature on Single-Peak Shear Mechanical Properties

**Figure 3:**
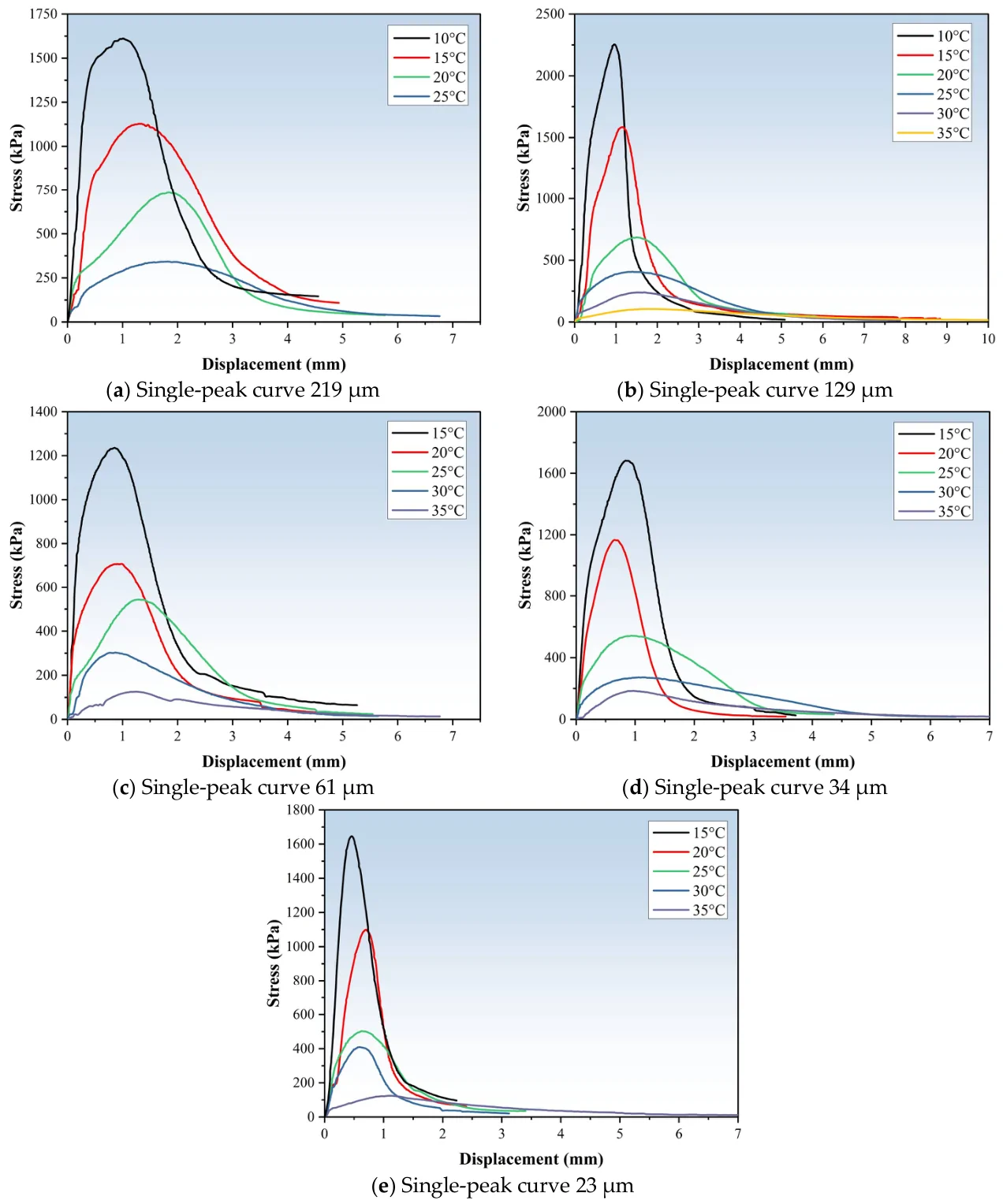
The influence curve of temperature on the shear mechanical properties of a single peak.

In the shear tests, a single peak was observed in all test groups; the typical single peak observed in each group is shown in Figure 3.

It is clearly evident that the characteristic of the single-peak curve is that, as shear displacement increases, the shear strength rises gradually until it reaches a peak, after which it begins to decrease; furthermore, the lower the temperature, the greater the peak shear strength. Consequently, lowering the temperature can enhance the shear strength at the asphalt–aggregate interface. The mean values and displacements for the single-peak curves are summarised from Table 4, Table 5, Table 6, Table 7 and Table 8.

**Figure 4:**
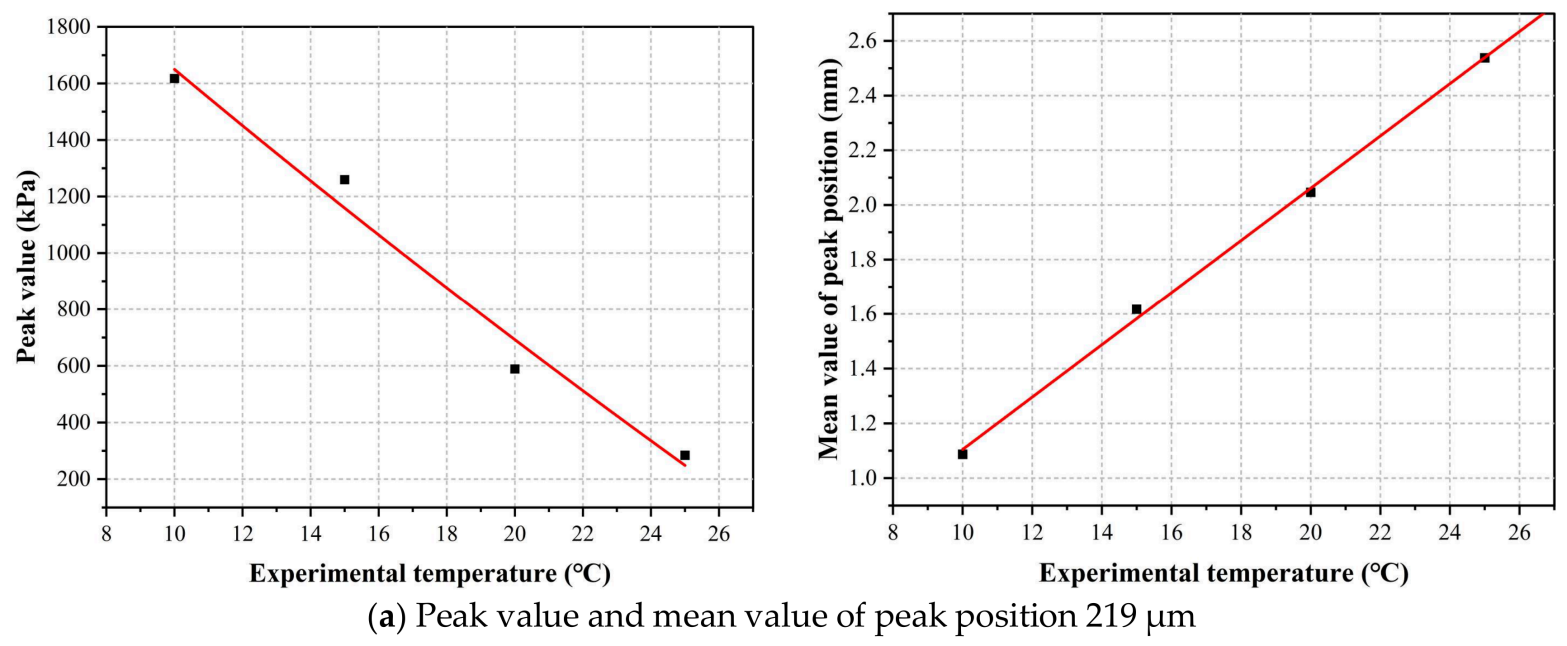

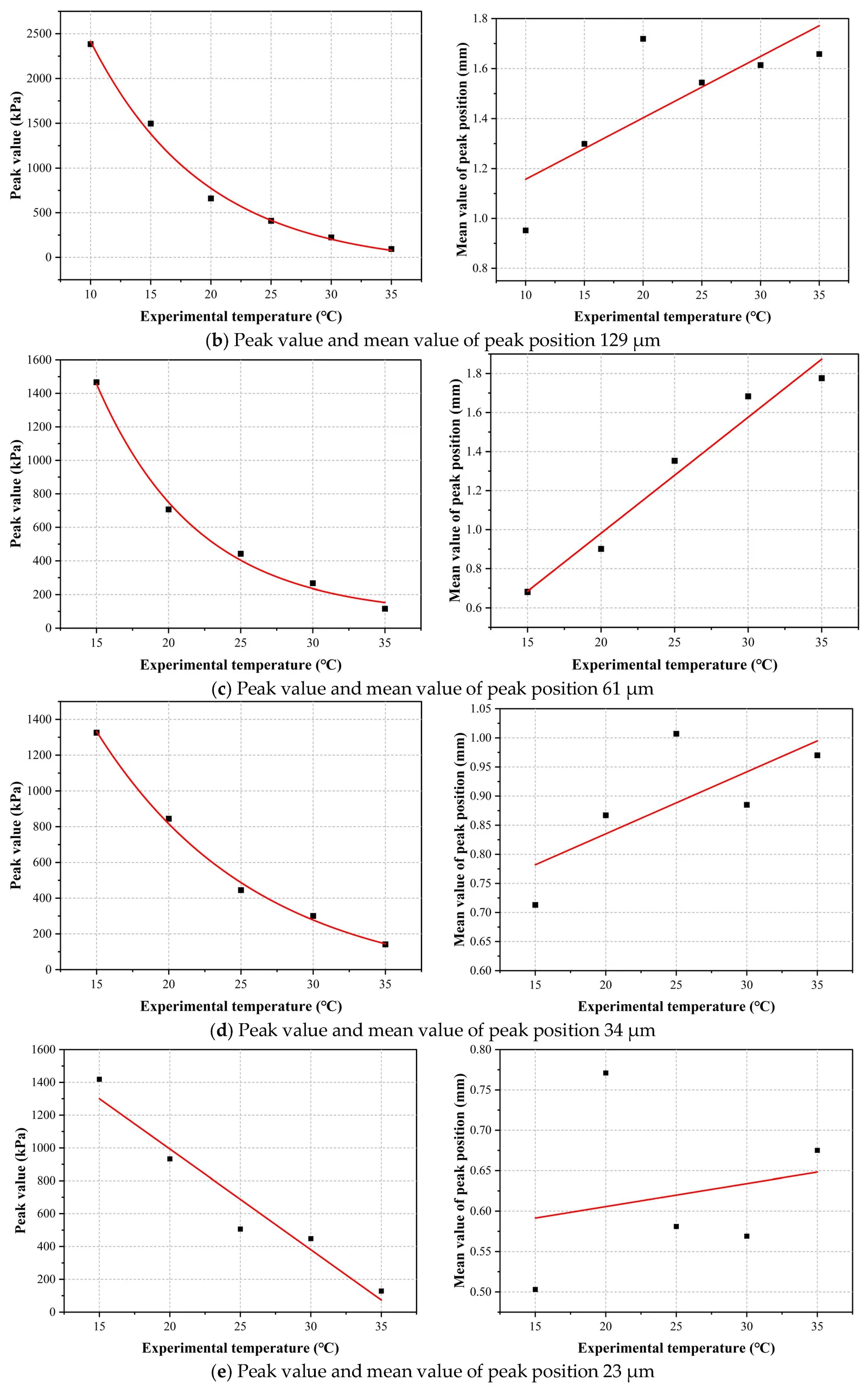
The Effect of temperature on shear strength and peak displacement.

From the Figure 4, experiments have shown that, as temperature increases, the shear strength at the asphalt-aggregate interface decreases rapidly at first and then more gradually, eventually approaching zero. The experiments indicate that at low temperatures, the bond strength at the asphalt-aggregate interface is high, and the shear failure mode at the interface is primarily adhesive failure; at high temperatures, the bond strength at the asphalt-aggregate interface is low, and the shear failure mode at the interface is primarily cohesive failure.

#### 3.1.2. Effect of Temperature on Single-Peak Shear Fracture Energy

**Figure 5:**
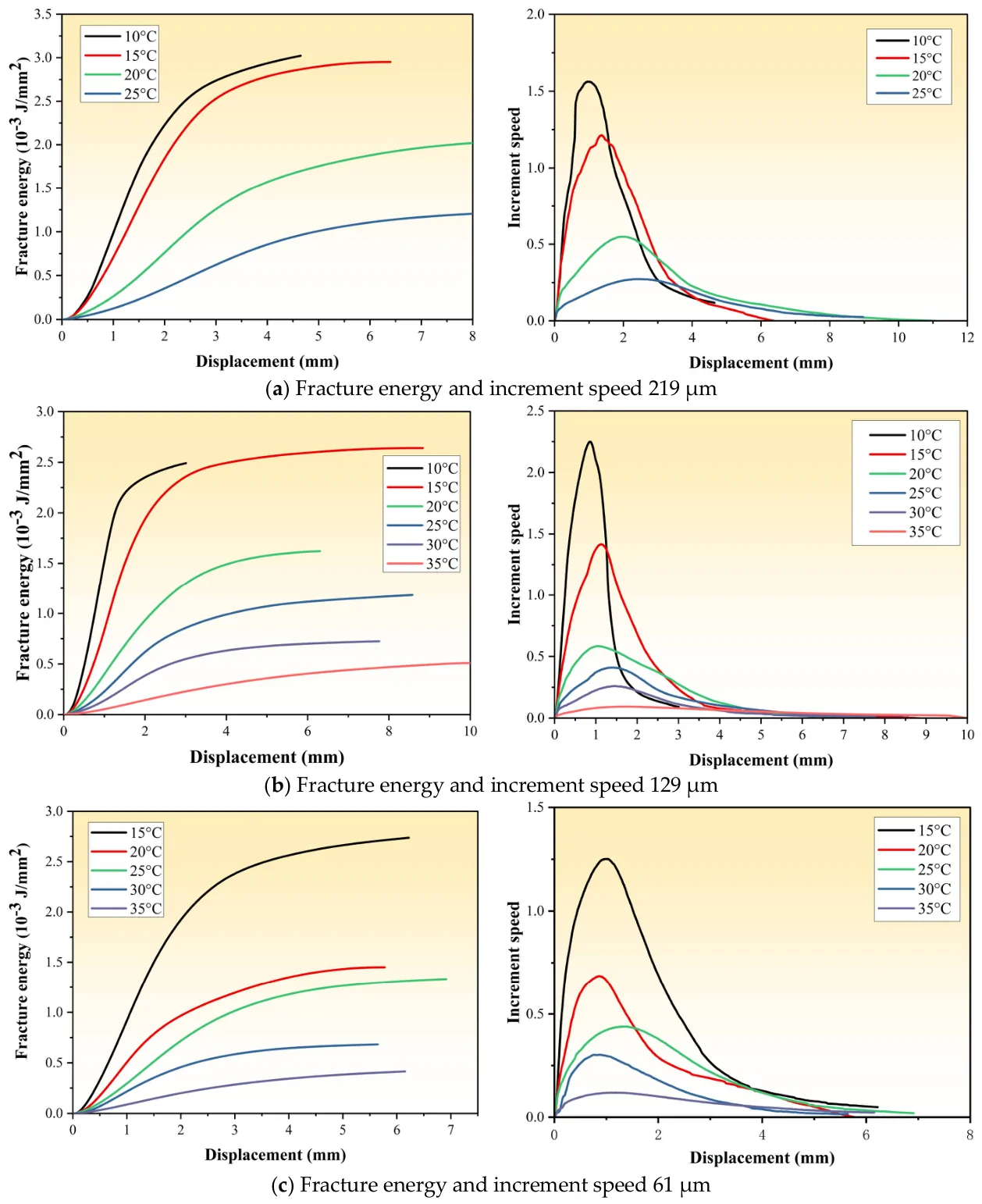

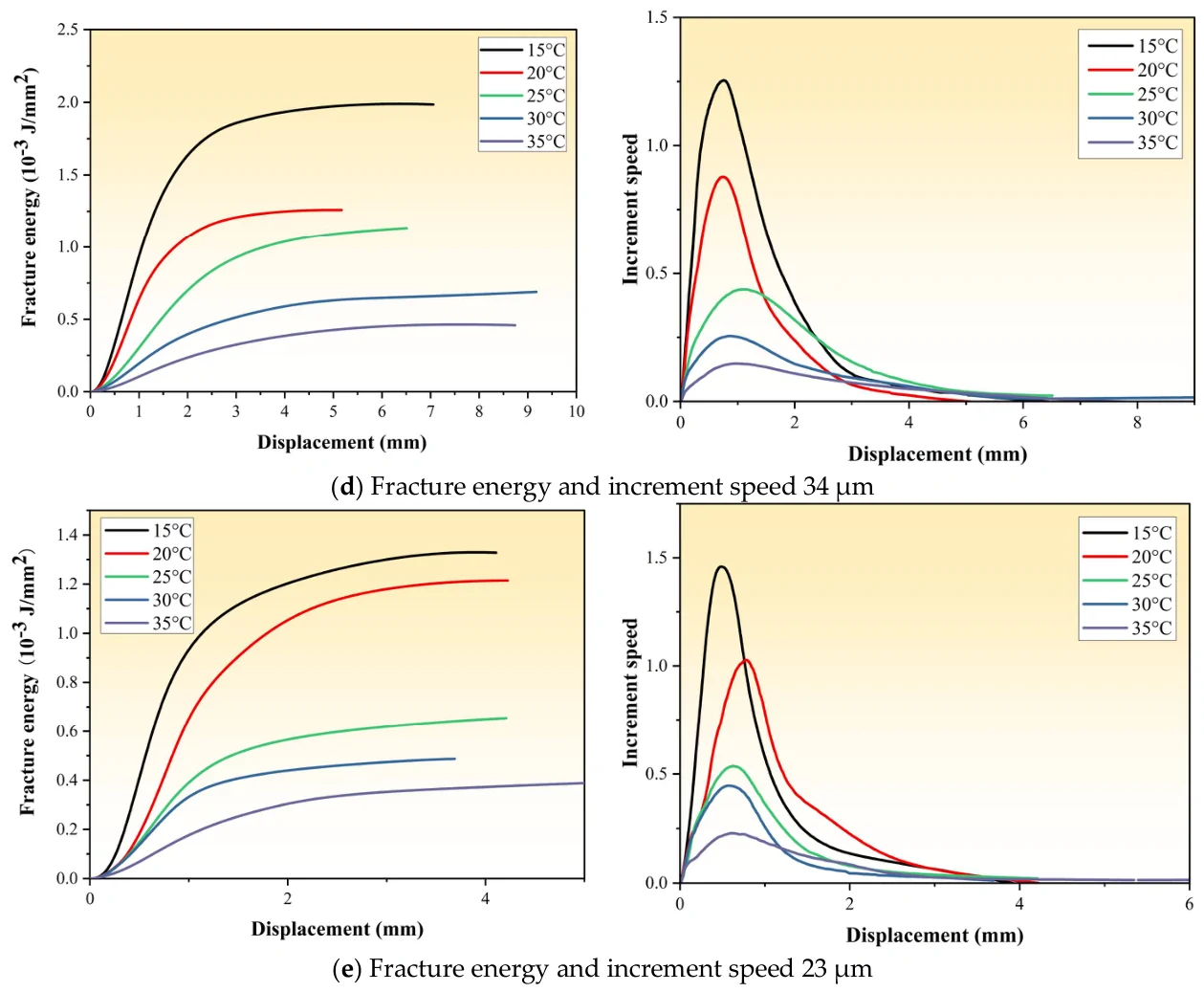
The effect of temperature on Single-Peak fracture energy and growth rate.

Figure 5 shows the single-peak shear fracture energy and the rate of increase (first derivative of the shear fracture energy) for asphalt films of different thicknesses at various temperatures.

The values assigned to fracture energy parameters are, in fact, those corresponding to interface failure; in shear tests at the asphalt–aggregate interface, it is not possible to determine whether interface failure has occurred simply by visual inspection. In such cases, a parameter is required to define the criterion for interface failure [28,29]. As shown in Table 9, Table 10, Table 11, Table 12 and Table 13, the displacement and fracture energy at which the rate of increase in shear fracture energy falls below a certain value can be defined as the ultimate displacement and maximum fracture energy of the shear specimen [30,31,32]. The displacement and fracture energy at which the rate of increase falls below 0.05 are taken as the ultimate displacement and maximum fracture energy.

Shear fracture energy is calculated as the energy dissipated per unit interfacial area during the failure process. For each specimen, the cumulative fracture energy G*_f_*(*d*) is obtained by integrating the shear force–displacement curve using the trapezoidal rule:

(1) $$G_{f}(d)\;=\;\frac{1}{A}\int_{0}^{d}P(s)\mathrm{ds}\approx \frac{1}{A}\sum_{i=1}^{N-1}\frac{P(S_{i})+P(S_{i+1})}{2}\cdot (S_{i+1}-S_{i})$$

where *P*(*s*) is the shear force at displacement s, A is the interfacial area of the specimen, and the summation is performed over the discrete force–displacement data points recorded during testing at a sampling rate. The resulting fracture energy is expressed in units of J/mm^2^.

The 0.05 threshold is adopted directly from the established methodology developed and validated for asphalt–aggregate shear fracture energy characterisation. It represents the optimal balance between capturing the full fracture energy and excluding post-failure artefacts, as determined through systematic sensitivity analysis on a large dataset of asphalt–aggregate shear tests. This criterion provides an objective and reproducible method for defining the endpoint of the fracture process, eliminating the subjectivity that would be introduced by visual inspection or arbitrary displacement cutoffs.

The single-peak shear fracture energy at the asphalt–aggregate interface is directly correlated with temperature. Under low-temperature conditions of 10 °C and 15 °C, with asphalt film thicknesses of 219 μm and 129 μm, respectively, the single-peak shear fracture energy at the asphalt–aggregate interface shows little difference; however, there is a clear difference in the single-peak shear strength and ultimate displacement at the two temperatures. Under these two asphalt film thickness conditions, the fracture energy dissipated at the interface at this point does not differ significantly, but the shear strength differs considerably. The viscoelastic behaviour exhibited by the asphalt–aggregate interface varies; at 15 °C, both the peak displacement and ultimate displacement are greater than at 10 °C, indicating that the viscoelasticity of the interface is stronger at this temperature. As the temperature rises, the single-peak shear fracture energy and shear strength at the asphalt–aggregate interface decrease markedly; when the asphalt film thickness is 61 μm, 34 μm and 23 μm, the single-peak shear fracture energy and shear strength at the asphalt–aggregate interface decrease with increasing temperature.

#### 3.1.3. Effect of Temperature on the Interface Properties of Asphalt and Aggregates

The following discussion presents mechanistic interpretations of the observed temperature-dependent interfacial behaviour. While the experimental data directly demonstrate the effects of temperature on shear strength and peak displacement, the mechanistic explanations discussed below are based on established literature and theoretical frameworks, rather than being directly measured in the present study. These interpretations are offered to provide a coherent understanding of the observed trends and to guide future investigations.

The properties of asphalt mixtures are determined by the adhesion between the asphalt and the aggregates. Asphalt plays a vital role as a binder, and its quality directly determines the performance of the asphalt–aggregate interface. Asphalt is a temperature-sensitive material; temperature has a significant impact on its properties, which in turn affects the performance of the asphalt–aggregate interface [33,34,35].

Asphalt generally comprises four components: saturated fractions, aromatic fractions, gum and bituminous matter. The higher the content of saturated and aromatic fractions, the lower the softening point of the asphalt; the gum directly determines the asphalt’s elongation and adhesive strength, whilst the bituminous matter determines its adhesive strength, viscosity and temperature stability. Through experiments examining changes in the composition of asphalt at different temperatures, researchers have found that as the temperature decreases, light fractions are converted into heavy fractions, and the oil fraction is converted into resin; the rate at which resin is converted into bituminous matter is faster than the rate at which the oil fraction is converted into resin. This indicates that the composition of asphalt also undergoes corresponding changes with variations in temperature [36,37].

The property whereby the viscosity and plasticity of asphalt vary with changes in temperature is known as the temperature sensitivity of asphalt, also referred to as temperature stability. Asphalt is an amorphous, non-crystalline polymeric compound whose mechanical properties are significantly influenced by temperature. When the temperature rises to a certain value, the asphalt softens and exhibits viscous behaviour; when the temperature falls to a certain value, the asphalt becomes brittle and exhibits plastic behaviour. Research indicates that the shear properties of asphalt mixtures are highly influenced by temperature; as temperature rises, the shear strength of asphalt mixtures decreases significantly, and the rate of decline is substantial. Corresponding to the results of the interface shear tests on asphalt-aggregate mixtures presented in this paper, the shear strength at the asphalt-aggregate interface is directly affected by temperature. As the temperature rises, the shear strength at the asphalt-aggregate interface decreases significantly, and the rate of decrease is very large. This indicates that the trend in the shear mechanical properties of the asphalt-aggregate interface with temperature corresponds to the trend in the shear mechanical properties of the asphalt mixture with temperature; therefore, research into the properties of the asphalt-aggregate interface can reflect the overall performance of the asphalt mixture [38,39].

Mechanical theory holds that the forces of interaction between asphalt and aggregates consist primarily of mechanical adhesion, van der Waals forces and hydrogen bonds. As the temperature gradually rises from low to high, the asphalt gradually transitions from an elastic state to a viscoelastic state, and finally to a viscous state. At this point, the viscoelasticity of the asphalt gradually decreases with rising temperature, resulting in a reduction in the interlocking force between the asphalt and the aggregate surfaces—that is, the mechanical adhesion. As the temperature rises, intermolecular motion intensifies and intermolecular forces weaken, resulting in a reduction in the van der Waals forces between the asphalt and the aggregates; the strength of hydrogen bonds gradually diminishes with increasing temperature, and at high temperatures, hydrogen bonds may even disappear entirely. Consequently, an increase in temperature reduces the intermolecular forces between the asphalt and the aggregates [40,41,42].

During the preparation of sandwich specimens, wetting occurs at the interface between the asphalt and the aggregate; the original solid–gas interface is replaced by a liquid–solid interface, at which point surface tension develops at the asphalt–aggregate interface. As the temperature rises, the surface tension at the interface decreases; when the temperature reaches the critical temperature T_0_ of the asphalt, the surface tension at the interface tends towards zero.

The effect of temperature on interfacial surface tension can be explained in two ways. Firstly, temperature affects the intermolecular forces within the liquid. As the temperature rises, the thermal motion of the molecules intensifies, their kinetic energy increases, and the intermolecular attractive forces weaken; consequently, the energy required for liquid molecules to move from the interior to the surface decreases. Secondly, changes in temperature affect the bulk densities on either side of the interface. As the temperature rises, the density difference between the two phases adjacent to the surface layer decreases; meanwhile, as the temperature increases, the density of the vapour phase decreases, and so does the attractive force exerted on the surface molecules, resulting in a decrease in surface tension. Macroscopically, both factors manifest as a decrease in interfacial surface tension as the temperature rises.

Consequently, at lower temperatures, as the elastic properties of asphalt improve, its cohesive strength increases. Although the adhesive strength at the asphalt–aggregate interface also increases, this interface is more susceptible to failure than the interior of the asphalt, and damage tends to originate at the interface; therefore, adhesive failure plays the predominant role; As the temperature gradually rises, the rheological properties of the asphalt improve, and failure may occur both within the asphalt and at the interface; when the temperature rises to a certain level, the asphalt becomes more fluid, and at this point, failure at the asphalt–aggregate interface is more likely to occur within the asphalt itself; consequently, failure at the asphalt–aggregate interface at this stage is primarily cohesive failure.

### 3.2. The Effect of Asphalt Film Thickness on the Shear Mechanical Properties at the Asphalt–Aggregate Interface

#### 3.2.1. The Effect of Asphalt Membrane Thickness on the Shear Mechanical Properties of a Single Crest

**Figure 6:**
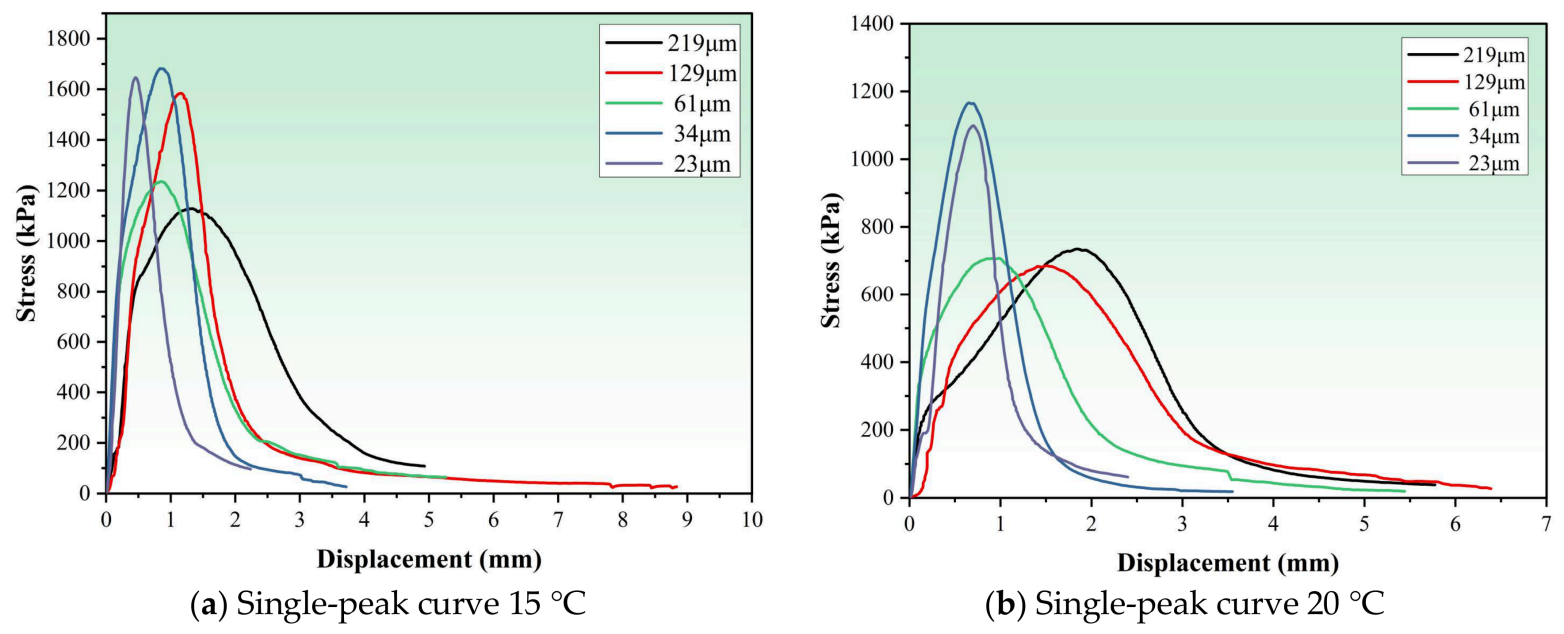

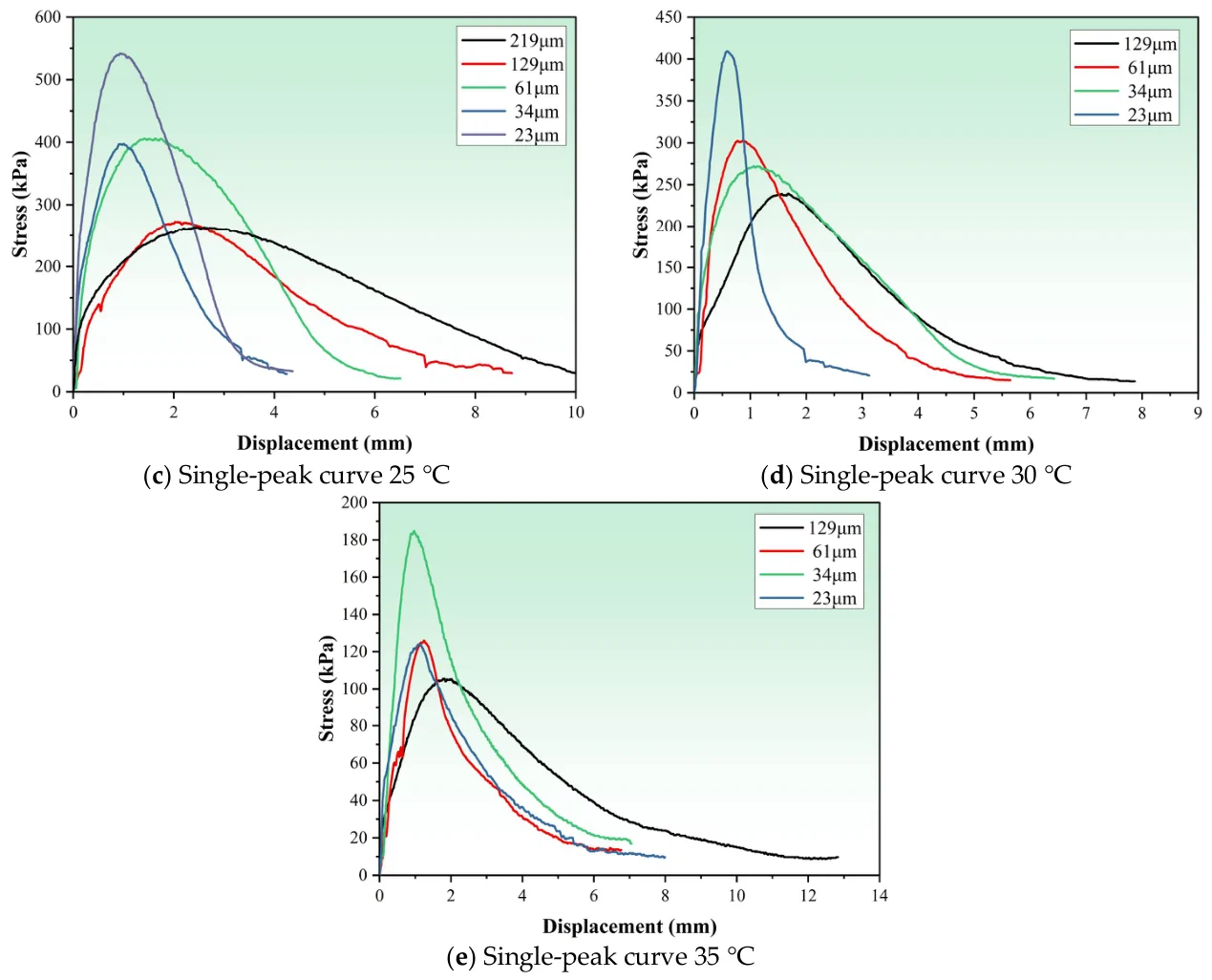
Single-wave peak curve for bituminous membrane thickness.

In the shear tests, a single peak was observed in all test groups; a typical single peak from each group is shown in Figure 6.

The statistics on the peak values and displacements of the single peaks observed in each group are shown in Table 14, Table 15, Table 16, Table 17 and Table 18.

**Figure 7:**
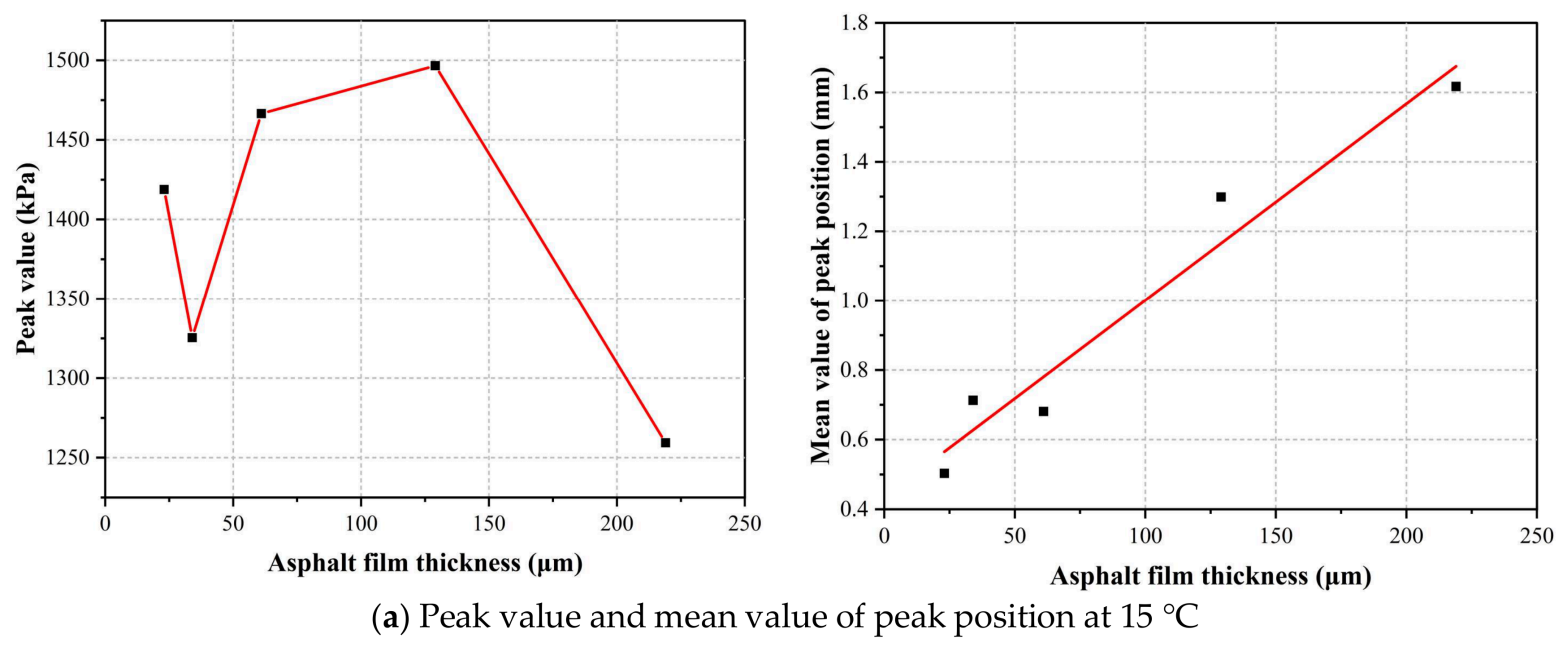

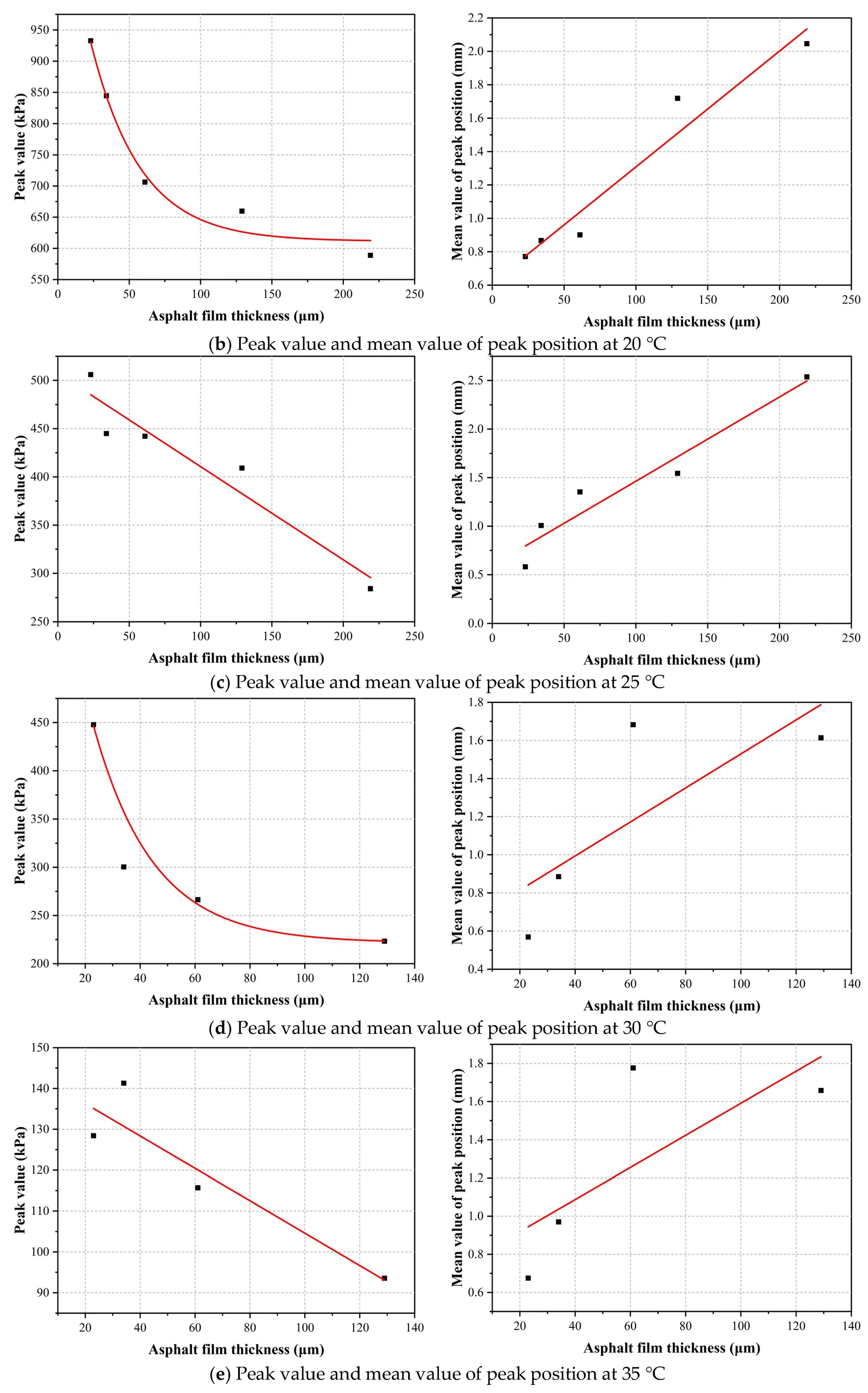
Shear strength and peak displacement of asphalt membranes as a function of thickness.

From the Figure 7, The tests revealed that, as the thickness of the asphalt film increased, the shear strength at the asphalt–aggregate interface decreased, whilst the position of the maximum shear stress gradually shifted upwards as the thickness of the asphalt film decreased. By calculating the mean peak value for the single peak observed under different asphalt film thickness conditions, it is possible to determine the pattern of how asphalt film thickness affects the adhesive properties at the asphalt–aggregate interface.

The tests revealed that there is a linear relationship between the thickness of the bituminous film and its strength: the thicker the bituminous film, the lower the shear strength at the asphalt-aggregate interface; conversely, the thinner the bituminous film, the higher the shear strength at the asphalt-aggregate interface.

#### 3.2.2. The Effect of Asphalt Film Thickness on the Shear Fracture Energy of a Single Peak

**Figure 8:**
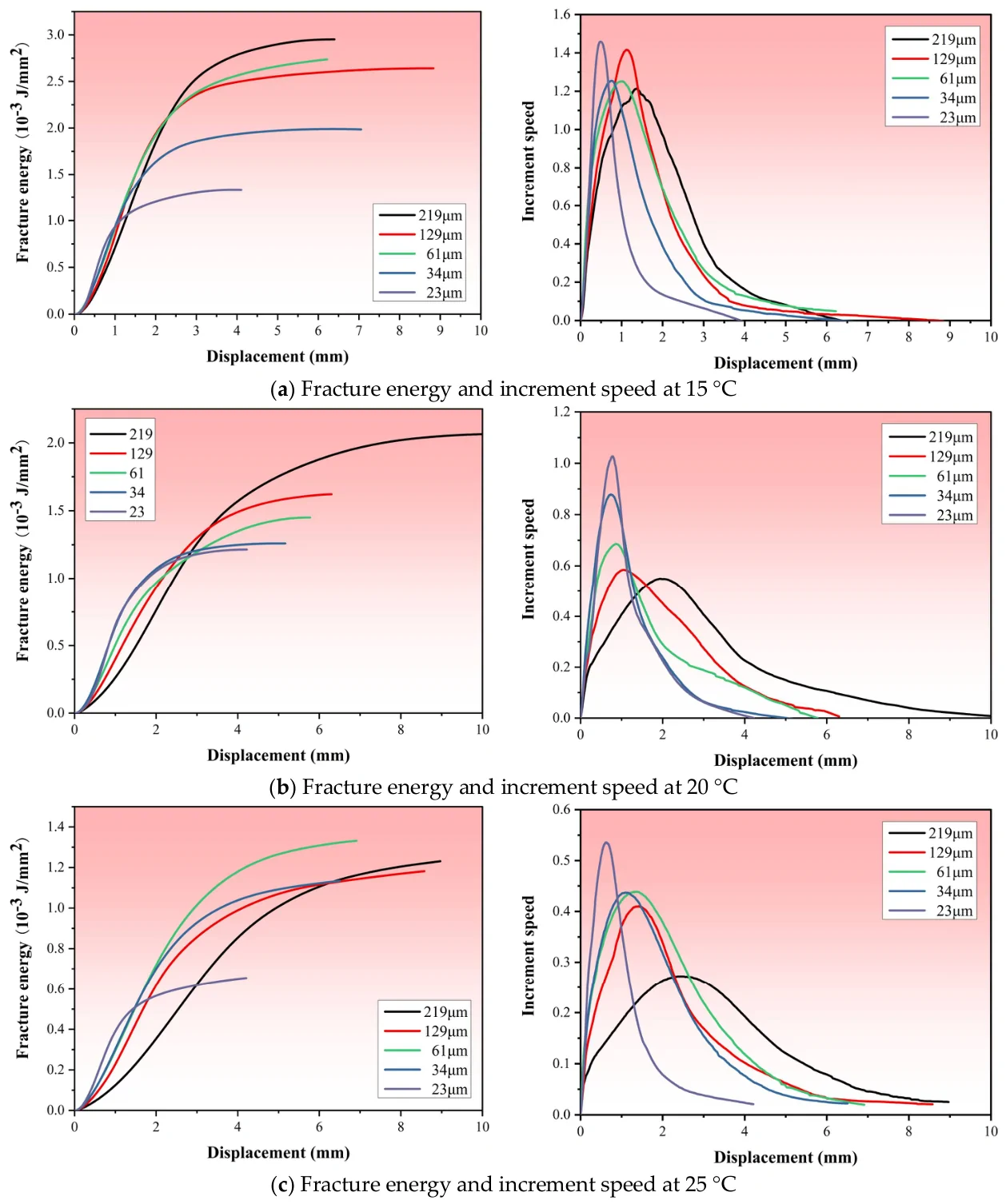

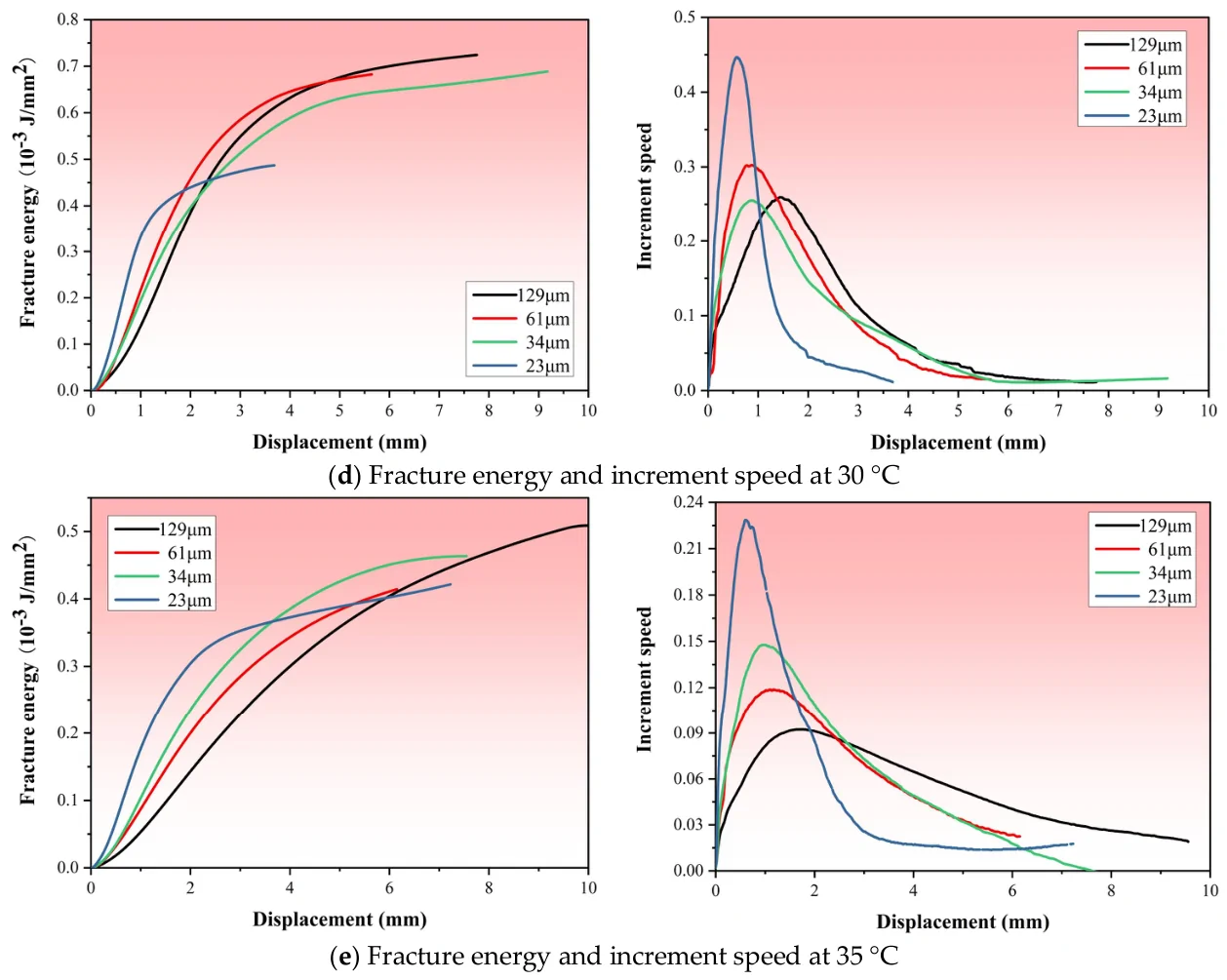
Single-peak fracture energy and growth rate.

Figure 8 shows the average shear fracture energy and growth rate of single-peak curves for different asphalt film thicknesses across the various temperature groups.

The maximum fracture energy and ultimate displacement for a single wave peak are shown in Table 19, Table 20, Table 21, Table 22 and Table 23.

The thickness of the bituminous film has a significant influence on the single-crest shear fracture energy at the asphalt–aggregate interface. At lower temperatures, when the asphalt film thickness ranges from 23 µm to 129 µm, regardless of temperature variations, the single-peak shear fracture energy at thicknesses of 129 µm and 61 µm is greater than that at thicknesses of 34 µm and 23 µm; furthermore, there is little difference in the single-peak shear fracture energy between 129 µm and 61 µm. In this case, the asphalt–aggregate interface exhibits good shear performance when the asphalt film thickness ranges from 61 µm to 129 µm. At higher temperatures, when the asphalt film thickness ranges from 23 µm to 129 µm, there is little variation in the single-crest shear fracture energy, and the shear failure mode at the asphalt–aggregate interface is primarily cohesive failure. When the asphalt film thickness is 219 µm, at lower temperatures, the single-peak shear fracture energy is greater than that at 129 µm and 61 µm. However, at higher temperatures, the shear fracture energy is lower than that at 129 µm and 61 µm, indicating that its shear performance is significantly influenced by temperature.

#### 3.2.3. Effect of Asphalt Film Thickness on the Shear Behaviour at the Asphalt-Aggregate Interface

The following discussion presents mechanistic interpretations of the observed film thickness-dependent behaviour. While the experimental data presented directly demonstrate the effects of film thickness on shear strength and fracture energy, the mechanistic explanations discussed below are based on established literature and theoretical frameworks, rather than being directly measured in the present study. These interpretations are offered to provide a coherent understanding of the observed trends.

The primary failure modes at the asphalt–aggregate interface are adhesion failure and cohesion failure. Adhesion failure occurs when asphalt detaches from the aggregate surface, resulting in interface failure, whilst cohesion failure refers to the separation of molecules within the asphalt film under load, leading to interface failure. The cohesive strength of structural asphalt is greater than that of free asphalt. The effect of asphalt film thickness on the asphalt–aggregate interface is primarily manifested as follows: when the asphalt film is relatively thin, the proportion of free asphalt at the interface is small, and failure within the asphalt is unlikely to occur; at which point failure at the asphalt-aggregate interface is more likely to originate from the bonding surface between the asphalt and the aggregate, and such failure manifests as adhesive failure [43,44].

As the thickness of the bituminous layer increases, the ratio of free asphalt at the asphalt-aggregate interface to the structural asphalt becomes appropriate; at this point, the failure at the asphalt-aggregate interface is shared by both components. When the bituminous film is relatively thick, the proportion of free asphalt at the asphalt-aggregate interface is higher, making the asphalt interior more susceptible to failure. In such cases, failure at the asphalt-aggregate interface is more likely to originate from within the asphalt, manifesting as cohesive failure.

Temperature also has a significant influence on the properties of free asphalt and structural asphalt; structural asphalt is formed on the surface of aggregates through processes such as chemical and physical adsorption. Both chemical and physical adsorption are influenced by temperature; a moderate increase in temperature favours chemical adsorption, whilst a decrease in temperature favours physical adsorption. Tests have shown that at low temperatures, the adhesive force between structural asphalt and the aggregate surface is weaker than the cohesive force of the structural asphalt itself; the shear failure mode of the asphalt-aggregate mixture is primarily adhesive failure, with interfacial failure occurring mainly at the aggregate surface; at high temperatures, the cohesive strength within the asphalt film is lower than the adhesive strength between the structural asphalt and the aggregate surface; the failure mode at the asphalt-aggregate interface is primarily cohesive failure, with interfacial failure occurring mainly within the asphalt itself.

The thickness of the bituminous film and temperature jointly influence the shear failure mode at the asphalt–aggregate interface. It should be noted that while the experimental data demonstrate a clear qualitative interdependence between temperature and film thickness, the film thickness-dependent failure mode transition temperature, and the temperature-dependent optimal film thickness for fracture energy. But we have not performed a formal statistical analysis to quantify this interaction. The term “coupled interaction” is therefore used to describe the observed qualitative interdependence rather than a statistically validated synergistic effect in the strict sense. Experiments have shown that at lower temperatures, the shear failure mode at the asphalt–aggregate interface is predominantly adhesive failure, with failure occurring mainly at the contact surface between the asphalt and the aggregate; at higher temperatures, the shear failure mode is predominantly cohesive failure, with failure occurring mainly within the asphalt itself; within a certain temperature range, the higher the temperature and the greater the thickness of the asphalt film, the greater the probability of cohesive failure; conversely, the lower the temperature and the thinner the asphalt film, the greater the probability of adhesive failure [45,46].

## 4. Conclusions

Through shear tests on asphalt-aggregate-asphalt sandwich specimens, this paper systematically investigates the coupled influence of temperature and asphalt film thickness on the shear mechanical behaviour at the asphalt-aggregate interface. Based on an analysis of the characteristics of stress–displacement curves, the evolution of fracture energy and failure modes, the following main conclusions were drawn:

(1) Temperature has a significant deteriorating effect on the shear strength at the asphalt–aggregate interface. As the temperature rises, the shear strength at the interface decreases rapidly at first and then more gradually, eventually approaching zero; the peak displacement increases overall, and the interface gradually transitions from brittle behaviour at low temperatures to viscous behaviour at high temperatures. The shear fracture energy decreases monotonically with rising temperature, with the rate of decrease being more pronounced in the low-temperature range, indicating that the load-bearing capacity of the interface is more sensitive to temperature changes at low temperatures.
(2) The effect of asphalt film thickness on interfacial shear strength follows a monotonically decreasing trend. As the asphalt film thickness increases, the interfacial shear strength decreases, whilst the peak displacement increases as the film thickness decreases; shear fracture energy remains at a relatively high level within the intermediate thickness range, whilst asphalt films that are either too thin or too thick are detrimental to the interfacial energy dissipation capacity.
(3) Temperature and the thickness of the bituminous film jointly determine the failure mode at the interface. Under low-temperature conditions, the cohesive strength of the asphalt is relatively high, and interface failure occurs primarily at the asphalt–aggregate interface, manifesting as adhesive failure; Under high-temperature conditions, the cohesive strength of the asphalt decreases significantly, and the failure path shifts to within the asphalt film, manifesting as cohesive failure. An increase in the thickness of the asphalt film promotes a transition towards cohesive failure, whilst a decrease in film thickness tends to favour adhesive failure; between these two extremes, a coupled and competitive relationship is observed.
(4) The temperature dependence of interfacial shear behaviour is consistent with mechanistic interpretations based on temperature-induced phase transitions in the bituminous components and the attenuation of interfacial forces, as described in the literature. Cooling is understood to promote the conversion of light components into heavier ones, thereby enhancing the cohesive strength of the asphalt; conversely, heating is understood to reduce intermolecular forces and interfacial surface tension, weakening the strength of both physical and chemical adsorption. These effects are consistent with an alteration in the distribution of interfacial weak points, which may drive a shift in the failure mode.
(5) The influence of asphalt film thickness on failure modes is consistent with the mechanistic interpretation that it regulates the ratio between bound asphalt and free asphalt, as proposed in the literature. When the film thickness is small, the proportion of bound asphalt is high, the interfacial adhesion strength is relatively weak, and the failure surface is located on the aggregate surface; as the film thickness increases, the proportion of free asphalt rises, the internal cohesion strength of the asphalt becomes the controlling factor, and the failure surface shifts to within the asphalt film. Rising temperatures are understood to alter the mechanical behaviour of both types of asphalt, further amplifying the effect of film thickness; together, these factors are interpreted to determine the ultimate failure path at the interface.

The conclusions of this study are based on a specific experimental framework: SBS-modified asphalt, limestone aggregate, controlled surface roughness, a single shear rate, dry and unaged conditions, and idealised sandwich specimens. These conditions may limit the direct quantitative generalizability of the findings to other material systems (e.g., base or alternative polymer-modified asphalts, acidic or recycled aggregates), test protocols (e.g., different loading rates), or field-relevant environmental factors (e.g., ageing and moisture). The mechanistic trends identified—particularly the coupled influence of temperature and film thickness on failure mode transitions—are expected to be qualitatively applicable to a broader range of asphalt–aggregate interfaces, but their quantitative values (e.g., critical transition temperatures, optimal film thickness ranges) require validation under expanded conditions. Future work should extend the experimental matrix to include varied binder types, aggregate mineralogies, surface textures, loading rates, ageing states, and moisture conditions to establish a more comprehensive framework.

The double-peak phenomenon was also observed during the tests; this is related to the bonding and adhesion mechanisms at the interface between the asphalt and the aggregate. This paper focuses solely on the single-peak phenomenon, analysing the patterns of change in the mechanical properties of the asphalt–aggregate interface at different temperatures and under different asphalt film thicknesses. Subsequent research may require a more in-depth analysis incorporating the double-peak phenomenon and the probabilities of their occurrence, in order to elucidate the mechanisms of bond failure and the patterns of damage evolution at the asphalt–aggregate interface.

The exclusion of double-peak curves does not introduce significant bias into the reported single-peak trends, as: (a) single-peak curves constitute the majority of specimens across all conditions; (b) the regions of highest double-peak frequency are precisely where the competitive transition between adhesion and cohesion is most pronounced, complementing rather than contradicting the single-peak findings; and (c) the exclusion criterion was applied consistently across all conditions. A dedicated analysis of the double-peak phenomenon, including the probability distribution of double-peak occurrence as a function of temperature and film thickness, is planned for future work.

## Figures and Tables

**Table 1:** Performance index of SBS-modified asphalt.

Items	Results	Specifications
Penetration (25 °C, 100 g, 5 s) (0.1 mm)	51	30–60
Softening point (°C)	83	≥60
Ductility (5 °C, 5 cm/min) (cm)	27	≥20
Density (15 °C) (g/cm^3^)	1.026	-

**Table 2:** Technical index of coarse aggregate [27].

Specifications	Los Angeles Abrasion Value (%)	Crushing Value of Stone (%)	Water Absorption (%)	Apparent Specific Gravity
Results	11.9	12.1	0.84	2.473

**Table 3:** Thickness of asphalt film.

Group	1	2	3	4	5
Weight (g)	200	700	1775	2519	5098
Asphalt film thickness (μm)	219	129	61	34	23

**Table 4:** Single-peak mean and displacement (219 μm).

Test Temperature (°C)	Peak Mean (kPa)	Standard Deviation (kPa)	Coefficient of Variation	Peak Displacement (mm)	Standard Deviation (mm)	Coefficient of Variation
10	1617.33	118.70	0.073	1.09	0.07	0.06
15	1259.34	136.61	0.108	1.62	0.21	0.131
20	588.84	87.27	0.148	2.05	0.31	0.151
25	284.14	28.79	0.101	2.54	0.16	0.063

**Table 5:** Single-peak mean and displacement (129 μm).

Test Temperature (°C)	Peak Mean (kPa)	Standard Deviation (kPa)	Coefficient of Variation	Peak Displacement (mm)	Standard Deviation (mm)	Coefficient of Variation
10	2383.84	149.44	0.063	0.95	0.14	0.143
15	1496.62	126.82	0.085	1.30	0.19	0.144
20	659.68	61.89	0.094	1.72	0.25	0.146
25	409.18	61.14	0.149	1.54	0.23	0.149
30	223.31	29.62	0.133	1.61	0.23	0.143
35	93.54	15.40	0.165	1.66	0.17	0.105

**Table 6:** Single-peak mean and displacement (61 μm).

Test Temperature (°C)	Peak Mean (kPa)	Standard Deviation (kPa)	Coefficient of Variation	Peak Displacement (mm)	Standard Deviation (mm)	Coefficient of Variation
15	1466.53	183.07	0.125	0.68	0.10	0.15
20	706.29	86.60	0.123	0.90	0.09	0.094
25	442.08	60.73	0.137	1.35	0.16	0.118
30	266.33	36.83	0.138	1.68	0.01	0.008
35	115.70	9.20	0.079	1.78	0.09	0.085

**Table 7:** Single-peak mean and displacement (34 μm).

Test Temperature (°C)	Peak Mean (kPa)	Standard Deviation (kPa)	Coefficient of Variation	Peak Displacement (mm)	Standard Deviation (mm)	Coefficient of Variation
15	1325.41	192.42	0.145	0.71	0.08	0.15
20	844.59	122.32	0.145	0.87	0.12	0.135
25	444.89	58.550	0.132	1.01	0.11	0.111
30	300.40	23.53	0.078	0.89	0.13	0.148
35	141.29	4.25	0.03	0.97	0.04	0.043

**Table 8:** Single-peak mean and displacement (23 μm).

Test Temperature (°C)	Peak Mean (kPa)	Standard Deviation (kPa)	Coefficient of Variation	Peak Displacement (mm)	Standard Deviation (mm)	Coefficient of Variation
15	1418.85	143.85	0.101	0.50	0.06	0.109
20	932.72	121.14	0.130	0.77	0.05	0.070
25	505.97	75.30	0.149	0.58	0.06	0.105
30	447.74	38.57	0.086	0.57	0.01	0.025
35	128.41	19.20	0.150	0.68	0.08	0.113

**Table 9:** Table of single-peak fracture energy parameters (219 μm).

Temperature (°C)	10	15	20	25
Maximum displacement (mm)	4.69	5.50	7.67	6.81
Maximum fracture energy (10^−3^ J/mm^2^)	3.03	2.93	2.01	1.16

**Table 10:** Table of single-peak fracture energy parameters (129 μm).

Temperature (°C)	10	15	20	25	30	35
Maximum displacement (mm)	3.01	4.90	5.17	5.36	4.22	5.17
Maximum fracture energy (10^−3^ J/mm^2^)	2.49	2.55	1.59	1.09	0.64	0.37

**Table 11:** Table of single-peak fracture energy parameters (61 μm).

Temperature (°C)	15	20	25	30	35
Maximum displacement (mm)	6.12	5.01	5.24	3.71	3.91
Maximum fracture energy (10^−3^ J/mm^2^)	2.73	1.43	1.28	0.63	0.34

**Table 12:** Table of single-peak fracture energy parameters (34 μm).

Temperature (°C)	15	20	25	30	35
Maximum displacement (mm)	4.10	3.26	4.56	4.24	3.95
Maximum fracture energy (10^−3^ J/mm^2^)	1.94	1.22	1.07	0.60	0.38

**Table 13:** Table of single-peak fracture energy parameters (23 μm).

Temperature (°C)	15	20	25	30	35
Maximum displacement (mm)	3.19	3.18	2.51	1.97	2.40
Maximum fracture energy (10^−3^ J/mm^2^)	1.31	1.19	0.60	0.44	0.33

**Table 14:** Single-peak mean and displacement (15 °C).

Asphalt Film Thickness (μm)	Peak Mean (kPa)	Standard Deviation (kPa)	Coefficient of Variation	Peak Displacement (mm)	Standard Deviation (mm)	Coefficient of Variation
219	1259.34	136.61	0.108	1.62	0.21	0.131
129	1496.62	126.82	0.085	1.30	0.18	0.144
61	1466.53	183.07	0.125	0.68	0.10	0.150
34	1325.41	192.42	0.145	0.71	0.08	0.150
23	1418.85	143.85	0.101	0.50	0.06	0.109

**Table 15:** Single-peak mean and displacement (20 °C).

Asphalt Film Thickness (μm)	Peak Mean (kPa)	Standard Deviation (kPa)	Coefficient of Variation	Peak Displacement (mm)	Standard Deviation (mm)	Coefficient of Variation
219	588.84	87.27	0.148	2.05	0.31	0.151
129	659.68	61.89	0.094	1.72	0.25	0.146
61	706.29	86.60	0.123	0.90	0.08	0.094
34	844.59	122.32	0.145	0.87	0.18	0.135
23	932.72	121.14	0.130	0.77	0.05	0.070

**Table 16:** Single-peak mean and displacement (25 °C).

Asphalt Film Thickness (μm)	Peak Mean (kPa)	Standard Deviation (kPa)	Coefficient of Variation	Peak Displacement (mm)	Standard Deviation (mm)	Coefficient of Variation
219	284.14	28.79	0.101	2.54	0.16	0.063
129	409.18	61.14	0.149	1.54	0.23	0.149
61	442.08	60.73	0.137	1.35	0.16	0.118
34	444.89	58.55	0.132	1.01	0.11	0.111
23	505.97	75.30	0.149	0.58	0.06	0.105

**Table 17:** Single-peak mean and displacement (30 °C).

Asphalt Film Thickness (μm)	Peak Mean (kPa)	Standard Deviation (kPa)	Coefficient of Variation	Peak Displacement (mm)	Standard Deviation (mm)	Coefficient of Variation
129	223.31	29.62	0.133	1.61	0.23	0.143
61	266.33	36.83	0.138	1.68	0.01	0.008
34	300.40	23.53	0.078	0.89	0.13	0.148
23	447.74	38.57	0.086	0.57	0.01	0.025

**Table 18:** Single-peak mean and displacement (35 °C).

Asphalt Film Thickness (μm)	Peak Mean (kPa)	Standard Deviation (kPa)	Coefficient of Variation	Peak Displacement (mm)	Standard Deviation (mm)	Coefficient of Variation
129	93.54	15.40	0.165	1.66	0.17	0.105
61	115.70	9.20	0.079	1.78	0.09	0.085
34	141.29	4.25	0.030	0.97	0.04	0.043
23	128.41	19.20	0.150	0.68	0.08	0.113

**Table 19:** Table of single-peak fracture energy parameters (15 °C).

Temperature (°C)	219	129	61	34	23
Maximum displacement (mm)	5.50	4.90	6.12	4.10	3.19
Maximum fracture energy (10^−3^ J/mm^2^)	2.93	2.55	2.73	1.94	1.31

**Table 20:** Table of single-peak fracture energy parameters (20 °C).

Temperature (°C)	219	129	61	34	23
Maximum displacement (mm)	7.67	5.17	5.01	3.26	3.18
Maximum fracture energy (10^−3^ J/mm^2^)	2.01	1.59	1.43	1.22	1.19

**Table 21:** Table of single-peak fracture energy parameters (25 °C).

Temperature (°C)	219	129	61	34	23
Maximum displacement (mm)	6.81	5.36	5.24	4.56	2.51
Maximum fracture energy (10^−3^ J/mm^2^)	1.16	1.09	1.28	1.07	0.60

**Table 22:** Table of single-peak fracture energy parameters (30 °C).

Temperature (°C)	129	61	34	23	129
Maximum displacement (mm)	4.22	3.17	4.24	1.97	4.22
Maximum fracture energy (10^−3^ J/mm^2^)	0.64	0.63	0.60	0.44	0.64

**Table 23:** Table of single-peak fracture energy parameters (35 °C).

Temperature (°C)	129	61	34	23
Maximum displacement (mm)	5.17	3.91	3.95	2.40
Maximum fracture energy (10^−3^ J/mm^2^)	0.37	0.34	0.38	0.33

## Data Availability

The original contributions presented in this study are included in the article. Further inquiries can be directed to the corresponding authors.
